# Upper Tract Urothelial Carcinoma: Molecular Pathogenesis and Current Treatment Strategies—A Narrative Review

**DOI:** 10.3390/cancers18152394

**Published:** 2026-07-25

**Authors:** Dominik Zawadzki, Natalia Libergal, Jaśmina Nowak, Hanna Grzanka, Maksymilian Mikołajczyk, Mikołaj Kisiała, Michał Tulski, Wojciech Krajewski, Tomasz Szydełko, Bartosz Małkiewicz

**Affiliations:** Department of Minimally Invasive and Robotic Urology, University Centre of Excellence in Urology, Wroclaw Medical University, 50-367 Wroclaw, Poland; natalia.libergal@student.umw.edu.pl (N.L.); jasmina.nowak@student.umw.edu.pl (J.N.); hanna.grzanka@student.umw.edu.pl (H.G.); maksymilian.mikolajczyk@student.umw.edu.pl (M.M.); mikolaj.kisiala@student.umw.edu.pl (M.K.); michal.tulski@student.umw.edu.pl (M.T.); bartosz.malkiewicz@umw.edu.pl (B.M.)

**Keywords:** upper tract urothelial carcinoma, molecular pathogenesis, risk factors, immunotherapy, targeted therapy, risk stratification

## Abstract

**Background**: Upper tract urothelial carcinoma (UTUC) is a rare malignancy representing approximately 5–10% of all urothelial cancers. Key risk factors include smoking, chemical exposures, selected metabolic conditions, and hereditary cancer syndromes. This narrative review summarises current knowledge on UTUC molecular pathogenesis, major risk determinants, and contemporary therapeutic strategies. **Methods**: This study was conducted as a narrative review with a structured literature search. PubMed, Web of Science, Embase, and Scopus were searched using predefined combinations of UTUC-related terms covering molecular pathogenesis, carcinogenic risk factors, and treatment strategies. The review was prepared according to SANRA principles to improve transparency and consistency; however, no formal systematic review methodology or meta-analysis was performed. **Results**: Available genomic studies indicate that UTUC has a molecular profile distinct from urothelial bladder carcinoma (UBC), with recurrent alterations involving FGFR3, HRAS, KMT2D, CDKN2A, KRAS, MYC, and BRIP1. Smoking, aristolochic acid exposure, Lynch syndrome, and possibly early-onset urolithiasis contribute to carcinogenesis through distinct but incompletely understood mechanisms. Surgical treatment remains the standard of care for high-risk localised disease, whereas perioperative chemotherapy, immunotherapy, and targeted agents are expanding treatment options, particularly in advanced disease. A substantial proportion of the therapeutic evidence, however, is derived from broader urothelial carcinoma populations rather than UTUC-specific studies. **Conclusions**: UTUC is biologically heterogeneous and shaped by both molecular alterations and environmental exposures. Although substantial progress has been made, important gaps remain in understanding UTUC-specific carcinogenic mechanisms and in defining evidence-based personalised treatment strategies. Better integration of molecular, environmental, and clinical data is needed to improve risk stratification and treatment selection.

## Simple Summary

Upper tract urothelial carcinoma is a rare and aggressive cancer of the urinary system that is often diagnosed at an advanced stage, making treatment challenging. This review explains how the disease develops through a combination of inherited genetic changes and environmental factors such as cigarette smoking, exposure to certain toxins, and hereditary cancer syndromes. It also summarises current treatment options, including surgery, chemotherapy, immunotherapy, and targeted therapies, while highlighting that many treatment recommendations are still based on studies of bladder cancer rather than this distinct disease. Recent discoveries show that upper tract urothelial carcinoma has unique biological characteristics that may require more personalised treatment approaches. Emerging strategies, such as blood-based biomarkers, molecular profiling, and the integration of multiple types of biological data, may improve diagnosis, treatment selection, and patient outcomes in the future. Greater investment in disease-specific clinical research is essential to develop more effective and individualised care for patients with this uncommon but serious cancer.

## 1. Introduction

Upper tract urothelial carcinoma (UTUC) is a malignancy that accounts for 5–10% of all urothelial cancers and 5–7% of all renal tumours [1,2]. Despite its rarity, approximately 66% of patients present with advanced, muscle-invasive disease at diagnosis [1]. Moreover, around 7% of cases are metastatic at presentation, 10–20% of new diagnoses are multifocal, and 20% have intercurrent urothelial bladder carcinoma (UBC). Localised UTUC is predominantly asymptomatic, which contributes to delayed detection [2]. Although UTUC and UBC share common risk factors and clinicopathological features, UTUC has distinct practical, biological, and clinical characteristics [2,3]. UTUC is diagnosed at an invasive stage considerably more frequently than UBC, affecting over 60% and 15–25% of cases, respectively [4]. This difference reflects both the unique anatomical characteristics of the upper urinary tract, including the thin or locally absent muscularis of the renal pelvis that promotes early tumour invasion and the diagnostic complexity of UTUC, particularly the difficulty in obtaining adequate biopsy specimens for accurate pathological staging and grading [5,6]. A comprehensive understanding of the epidemiology, clinical features, and high-risk populations, combined with integration of environmental and molecular data, is essential to improve risk stratification, develop preventive strategies, and advance personalised cancer management [1,7].

UTUC predominantly affects elderly patients, especially men aged 68–72-years [8]. At diagnosis, it is located twice as commonly in the renal pelvis as in the ureter [2]. Established risk factors include tobacco smoking, chemical exposures, alcohol consumption, obesity, and dyslipidaemia. Genetic predispositions, such as Lynch syndrome, and region-specific exposures, including aristolochic acid consumption in East Asia, also contribute substantially to carcinogenesis [8,9].

The management of UTUC remains challenging owing to its biological heterogeneity, the need to balance oncologic control with preservation of renal function, and the limitations of current diagnostic and staging tools [10,11,12]. Treatment strategies have evolved considerably in recent years, incorporating advances in surgical techniques, systemic therapies, immunotherapy, and molecularly targeted approaches, which are expanding therapeutic options, particularly in advanced disease [11,13,14,15].

The aim of this narrative review is to summarise current knowledge on the pathogenesis of UTUC and the impact of the most crucial risk factors on its carcinogenesis. Another objective is to provide a comprehensive overview of currently available and emerging therapeutic approaches.

## 2. Materials and Methods

This study was conducted as a narrative review with a structured literature search. The review was prepared with reference to the SANRA (Scale for the Assessment of Narrative Review Articles) checklist in order to improve transparency, consistency, and reporting quality [16]. It should be emphasised that this was not a systematic review and no formal meta-analysis was performed.

The literature search was conducted using PubMed, Web of Science, Embase, and Scopus (Table 1). The search strategy was based on combinations of the following key terms: ‘upper tract urothelial carcinoma’ AND ‘molecular pathogenesis’, ‘upper tract urothelial carcinoma’ AND ‘treatment’, ‘UTUC’ AND ‘smoking’, ‘UTUC’ AND ‘Lynch syndrome’, ‘UTUC’ AND ‘aristolochic acid’, ‘UTUC’ AND ‘molecular targets’, and ‘UTUC’ AND ‘management’. Additional focused searches were performed for selected mechanisms of carcinogenesis and emerging therapeutic approaches.

Titles and abstracts were independently screened by two reviewers. Full texts were assessed whenever available; when full texts were unavailable, detailed abstracts were evaluated. Articles were included if they addressed UTUC pathogenesis, carcinogenic risk factors, molecular background, or treatment strategies. Conference abstracts, papers without extractable or relevant data, and studies published in languages not accessible to the authors were excluded.

After removal of duplicates and application of the eligibility criteria, 71 publications were included in the final review, comprising original studies, systematic reviews, and comparative clinical studies. For clarity of presentation, the evidence was grouped into five thematic categories: molecular pathogenesis, field cancerization versus monoclonal origin, smoking-related urothelial carcinogenesis, urothelial carcinogenesis associated with other factors, and UTUC management.

## 3. Molecular Pathogenesis

Recent scientific evidence highlights notable differences between the molecular carcinogenesis of UTUC and that of UBC [17]. Although data on the molecular pathogenesis of UTUC remain limited, several genomic studies have clarified important aspects [3].

**Figure 1:**
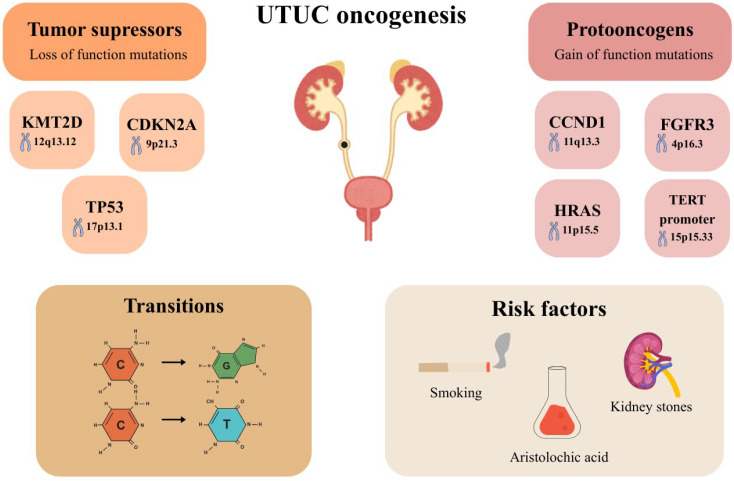
UTUC oncogenesis: Examples of genes that often become hot spots for mutations in UTUC. Panel in the lower left corner: Transitions between cytosine and guanine or cytosine and thiamine are more common in UTUC than in UBC. Panel in the lower right corner: Most common risk factors of UTUC include: smoking, aristolochic acid and kidney stones.

Fujii et al. analysed 199 fresh-frozen tumour samples and identified 51,709 mutations, demonstrating variation in mutation frequency between UTUC and UBC as well as be- tween tumours arising in the ureter and renal pelvis. Compared with UBC, UTUC showed a smaller mutational burden, averaging 5.8 mutations per megabase [3]. Robinson et al. confirmed a lower overall mutational burden in high-grade UTUC relative to UCB using RNA sequencing and whole-exome sequencing [18]. In UTUC, single nucleotide variants were dominated by C>T and C>G transitions. Notably, KMT2D mutations were considerably more frequent in ureteral than in bladder cancers (85% vs. 25%), while CDKN2A mutations were preferentially found in UTUC and ERBB2 mutations were more common in UBC. Anatomical location also influenced mutational patterns: ureteral tumours showed higher frequencies of KMT2D and TP53 mutations, whereas HRAS, KDM6A, and TERT promoter mutations were more prevalent in pelvic tumours. Regarding invasiveness, TP53 and CCND1 mutations were more common in invasive tumours, while FGFR3, HRAS, and TERT promoter mutations predominated in non-invasive disease. A RAS-mutated subtype, restricted to the renal pelvis and accounting for 15.1% of cases, was characterised by frequent DDX17 and TERT promoter mutations, chromosomal copy number alterations, and an aggressive histology with intermediate prognosis [3]. Yuen et al. found that MT2D, BRIP1, CDKN2B, KRAS, MYC and FGFR3 alterations were more frequent in UTUC than in UBC [19], and Audenet et al. reported a higher frequency of HRAS mutation and a lower frequency of TP53 mutation in UTUC compared with UBC [20] (Figure 1) (Table 2).

Based on these molecular characteristics, UTUCs are classified into five subtypes: hypermutated, TP53/MDM2-mutated, RAS-mutated, FGFR3-mutated, and triple-negative—a classification with clinical relevance regarding histological features and prognostic outcomes [3]. The FGFR3-mutated subtype is characterised by a low tumour mutational bur- den (TMB) and a T-cell-depleted microenvironment, resulting in an immunologically ‘cold’ phenotype with reduced PD-L1 expression [18,21]. Additionally, the T/T rs9642880 genotype has been identified as both a susceptibility factor and a marker of disease aggressiveness [21].

Overall, currently available molecular data suggest that UTUC is biologically distinct from UBC, particularly with regard to the frequency of FGFR3, HRAS, KMT2D, CDKN2A, KRAS, MYC, and BRIP1 alterations and site-specific mutation patterns. However, the number of dedicated UTUC genomic studies remains limited, and the clinical implications of many of these molecular differences are still being defined. The current evidence supports biological distinctiveness, but there is not yet a fully mature molecular classification ready for broad routine clinical implementation.

**Table 2:** Impact of molecular changes on the characteristics of urothelial carcinoma, including tumour localisation and invasiveness.

Characteristic	Molecular Changes	Implication	Reference
**Localization**			
**UTUC (general)**	C>T and C>G transitions; KMT2D, CDKN2A, BRIP1, CDKN2B, KRAS, MYC, FGFR3, HRAS mutations	Molecular profile distinct from UBC	[3,19,20]
**Ureter**	KMT2D and TP53 mutation	Site-specific mutational pattern	[3]
**Renal pelvis**	DDX17 and TERT promoter mutations; chromosomal copy number alterations	Site-specific mutational pattern	[3]
**UBC**	ERBB2 mutation	Differentiates UBC from UTUC	[3]
**Invasiveness**			
**Invasive UTUC**	TP53 and CCND1 mutation	Associated with disease progression	[3]
**Non-invasive UTUC**	FGFR3, HRAS and TERT promoter mutation	Associated with lower-stage disease	[3]
**UTUC risk factor**	T/T rs9642880 genotype	Susceptibility and aggressive disease	[22]

Abbreviations: UTUC, upper tract urothelial carcinoma; UBC, urothelial bladder carcinoma.

## 4. Field Cancerization vs. Monoclonal Origin

During nephrectomy performed for UTUC, approximately one-third of the distal ureter is left in situ. Kakizoe et al. found that urothelial carcinomas develop in the remaining ureteral segment in approximately 20–50% of patients and that 15–50% of patients who undergo total nephroureterectomy subsequently develop bladder carcinoma [23]. These observations highlight the multifocal nature of urothelial carcinogenesis, which can be explained by two main models: field cancerization and clonal spread.

The field cancerization model posits that the carcinogen-exposed urothelium undergoes multicentric malignant transformation, resulting in tumours that arise independently at different sites. The monoclonal model, in contrast, assumes that all tumours derive from a single transformed precursor cell that disseminates via intraluminal seeding or direct extension.

Most currently available molecular studies support a predominantly monoclonal origin in synchronous or metachronous urothelial tumours involving the upper tract and bladder. Van Doeveren et al., in a systematic review of five studies encompassing 14 patients with paired upper tract and bladder tumours, found that 85.1% of tumours were of monoclonal origin [24]. Similarly, in patients with primary UTUC and synchronous or metachronous intravesical recurrence, 93.5% exhibited concordant molecular alterations between paired tumours, further supporting shared clonal ancestry [24]. Consistent with this, Sidransky et al. demonstrated identical X-chromosome inactivation patterns across multiple tumour sites within individual patients, confirming single-cellular ancestry [25]. Takahashi et al. also found discordant microsatellite alterations in only one of 14 UTUC patients who developed intravesical recurrence [26].

Some evidence, however, supports an element of polyclonality. Among 42 patients with paired bladder and upper tract tumours, 11 exhibited discordant TP53 mutations, while 2 had mutations in only a single tumour focus—findings that are consistent with independent tumour development [27]. Notably, as TP53 alterations typically arise late in urothelial carcinogenesis, mutational discordance may reflect subclonal divergence from a common progenitor rather than truly independent origins, and this should be considered when interpreting these results.

In summary, the available evidence favours monoclonal expansion as the dominant mechanism in many multifocal urothelial tumours, while field cancerization cannot be excluded. The relative contribution of each mechanism likely varies between patients and remains an area requiring further study.

## 5. Smoking-Induced Urothelial Carcinogenesis

Because direct mechanistic data for UTUC remain limited, part of the following discussion is based on evidence derived from bladder urothelial carcinoma and the broader urothelial carcinoma literature. Where possible, UTUC-specific findings are highlighted separately.

Tobacco smoking is a well-established risk factor for UTUC [28,29]. Epidemiologic studies consistently demonstrate increased UTUC risk among smokers, with a dose–response relationship observed for smoking intensity, duration, and cumulative exposure [30,31,32]. Depending on co-existing variables, the overall increase in carcinoma risk has been estimated at 2.7- to 7-fold [9]. Although UTUC is approximately twice as common in men [9], sex-specific differences have been observed in outcomes: female smokers—particularly heavy, long-term smokers—experience higher rates of recurrence and cancer-specific mortality [33]. Smoking has also been associated with worse oncological outcomes after radical nephroureterectomy (RNU), including increased recurrence and cancer-specific mortality with effects that follow a clear exposure–response relationship [34,35].

From a mechanistic perspective, tobacco smoke contains over 60 identified carcinogens, including polycyclic aromatic hydrocarbons, nitrosamines, and aromatic amines [8,28,36]. Among these, 4-aminobiphenyl (4-ABP) is considered one of the primary human urothelial carcinogens. Following metabolic activation via CYP1A2-mediated N-oxidation and conjugation, 4-ABP can form N-(deoxyguanosine-8-yl)-4-ABP adducts in urothelial cells, contributing to mutagenesis and malignant transformation [28,36,37]. Although much of the mechanistic evidence derives from bladder cancer models, these pathways are biologically relevant to UTUC.

Available UTUC-specific data suggest that smoking may also promote a more aggressive tumour phenotype. Smoking history has been correlated with adverse pathological features and worse cancer-specific outcomes, supporting its role as an independent prognostic factor [28,38,39]. Translational data further indicate that smoking may enhance tumour invasiveness by increasing intratumoural lymphatic vessel density and upregulating pro-metastatic mediators such as VEGF-D, COX-2, and MMP-9 [40]. However, these findings should be interpreted cautiously given the small number of dedicated UTUC studies.

Smoking cessation should be encouraged as both a primary and secondary preventive strategy. Evidence suggests that patients who quit smoking at least 10 years before RNU may have intravesical recurrence risks comparable to those of never-smokers [39] and that long-term former smokers experience fewer adverse oncological effects than current smokers [34]. Given the dose-dependent relationship between smoking and recurrence risk, early cessation is particularly important [35,41]. The impact of recent cessation on outcomes requires further investigation [35].

### Experimental Studies

Tissue-specific mutagenesis by N-butyl-N-(4-hydroxybutyl)nitrosamine (a tobacco-smoke nitrosamine) has been experimentally observed in the mouse bladder, but no appreciable mutagenesis was detected in the kidney, ureter, liver, or forestomach [42], suggesting that this compound’s mutagenic activity does not extend to upper urinary tract tissue under the conditions tested. In a next-generation sequencing study using the MSK-IMPACT assay, the most frequently altered genes in smokers with urothelial carcinoma were TERT promoter (55%), TP53 (43%), KDM6A (30%), and FGFR3 (27%), with a similar distribution in never-smokers [43]. Micronucleus assay data further support a mutagenic effect of smoking in exfoliated urothelial cells: micronucleated cell frequencies were significantly higher in current smokers (1.09%) and ex-smokers (0.95%) than in never-smokers (0.24%), with smoking being the only variable reaching statistical significance (*p* = 0.007) [44,45]. Individuals carrying NAT2 and GSTM1 polymorphisms appear to be at additional risk [46], consistent with the role of these metabolic variants in carcinogen detoxification. Overall, while experimental evidence confirms the mutagenic potential of tobacco-derived compounds in urothelial tissue, dedicated UTUC mechanistic studies remain scarce.

## 6. Mechanisms of Urothelial Carcinogenesis Associated with Other Factors

Gene–environment interactions play an important role in the risk of UTUC [7]. Several additional exposures have been implicated, including aristolochic acid (AA), urolithiasis, and hereditary cancer syndromes.

**Aristolochic acid.** Aristolochic acid, a component of certain herbal medicines, is an established UTUC carcinogen strongly associated with Balkan nephropathy and Chinese herb nephropathy [47]. Genome-wide analyses of AA-associated urothelial carcinomas have revealed an exceptionally high somatic mutation burden (~150 mutations/Mb), attributable to the ability of AA to form DNA adducts [48]. Fuji et al. further reported that a higher frequency of the hypomorphic ALDH2 allele, combined with AA exposure, contributes to UTUC risk in Asian patients [3]. At the molecular level, AA-exposed cells demonstrate overexpression of MMP-2 and MMP-9, increased urokinase-type plasminogen activator (uPA) activity, and activation of MAPK signalling via p-ERK and p-p38, collectively promoting cell migration and invasion [49]. Concurrently, tissue inhibitors TIMP-1 and TIMP-2 are downregulated, further facilitating invasive behaviour [49].

**Kidney stones.** Early-onset urolithiasis (diagnosis before age 40) has been identified as a risk factor for UTUC, although the association is not fully established [50]. Proposed mechanisms include epithelial irritation and injury leading to chronic inflammation and oxidative stress [50,51]. Concurrent urinary tract infections may contribute via bacterial genotoxins; the carcinogenic potential of colibactin, produced by uropathogenic *E. coli*, has been demonstrated in the bladder [52], although its role in UTUC carcinogenesis remains speculative. It is also possible that the observed association partly reflects shared risk factors such as obesity and diabetes mellitus [53].

**Lynch syndrome.** Lynch syndrome (LS) is a hereditary cause of elevated UTUC risk, with UTUC representing the third most common malignancy in LS patients and carrying an estimated 20% lifetime risk [17,54]. Studies suggest that 2.6–4.3% of all UTUC cases may be LS-associated [54]. In contrast to colorectal and endometrial cancer—where mismatch repair (MMR) immunohistochemistry (IHC) is routinely applied—systematic MMR IHC screening is not yet standard practice in UTUC, despite calls from several groups [54]. Current AUA and EAU guidelines (2024) recommend identifying patients at risk of LS and performing universal histological testing in those with a high prior probability of LS-related cancer [55]. Clinical indicators for germline testing in sporadic UTUC include age under 60 years, family history of UTUC, or first-degree relatives with Lynch-spectrum cancers [55] (Table 3).

## 7. UTUC Management

### 7.1. Risk Stratification

The management of UTUC begins with risk stratification into low- and high-risk categories as recommended by contemporary guidelines [10,11,12]. This classification plays a crucial role in determining appropriate management strategies and relies on an integrated assessment of clinical, endoscopic, cytological, and radiological data.

Key diagnostic modalities include cross-sectional imaging (CT/MRI), cystoscopy, ureteroscopy with biopsy, and urine cytology, all of which contribute to accurate preoperative assessment [10,11,12]. Risk stratification incorporates tumour grade, stage (≥T2), size, focality, and radiologic indicators such as local invasion and hydronephrosis [10,11,56]. Both the European Association of Urology (EAU) and the American Urological Association (AUA) guidelines provide structured risk models to guide clinical decision-making [10,12], and recent advances in endoscopic instrumentation and biopsy techniques have further improved diagnostic accuracy [12].

### 7.2. Radical Nephroureterectomy and Kidney-Sparing Surgery

Radical nephroureterectomy (RNU) with bladder cuff excision remains the gold standard for high-risk UTUC [10,11,56,57,58,59]. However, it is associated with considerable morbidity, including perioperative complications and decline in renal function, which is especially relevant in elderly patients [57].

Kidney-sparing surgery (KSS) has emerged as an alternative for patients with low-risk UTUC and for selected high-risk cases in whom renal preservation is imperative (single kidney, renal insufficiency, or bilateral tumours) [10]. KSS offers reduced morbidity and preserved renal function [10,57,59]. In appropriately selected patients, oncological outcomes in terms of cancer-specific survival are comparable to RNU; however, KSS is associated with higher recurrence rates and risk of progression requiring salvage RNU [57].

### 7.3. Adjuvant Chemotherapy

Adjuvant platinum-based chemotherapy has demonstrated a clear benefit in patients with high-risk or locally advanced UTUC [12,13,56]. It allows treatment to be tailored based on accurate pathological staging from RNU specimens, thereby reducing overtreatment in non-invasive disease. Leow et al. reported significant improvement in overall survival (OS, HR 0.43, 95% CI 0.21–0.89) and disease-free survival (DFS, HR 0.49, 95% CI 0.24–0.99) with adjuvant cisplatin versus surveillance [58].

The landmark POUT trial (NCT01993979)—a phase III randomised controlled study—enrolled patients with locally advanced or node-positive UTUC after RNU and demonstrated a significant DFS benefit at three years (71% vs. 46%, HR 0.45) with four cycles of gemcitabine plus cisplatin or carboplatin (the latter for GFR < 50 mL/min) [11,12,13,56]. Updated five-year data confirmed continued DFS benefit (62% vs. 45%, HR 0.55) with a non-significant trend towards improved OS (HR 0.6, *p* = 0.049) [12,13]. Cisplatin-based therapy remains preferred for eligible patients; carboplatin, although frequently substituted in patients with impaired renal function, appears less effective (DFS HR 0.66 vs. 0.35 for cisplatin in the POUT study) [56]. Only approximately 20% of patients maintain sufficient renal function (GFR ≥ 60 mL/min) to receive cisplatin after RNU, highlighting the importance of renal function monitoring [11].

### 7.4. Neoadjuvant Chemotherapy

Neoadjuvant chemotherapy (NAC) is recommended in selected high-risk patients, particularly when postoperative renal function decline is anticipated. Because both kidneys are retained prior to surgery, cisplatin eligibility is higher in the neoadjuvant compared with the adjuvant setting [13,56,58]. Trials such as ECOG A8141 and NCT01261728 reported approximately 60% downstaging and 14–19% complete response rates, while cisplatin–gemcitabine regimens demonstrated 63% response and 19% complete response with favourable survival outcomes [13,58]. Meta-analyses indicate improved OS and a higher likelihood of node-negative disease after NAC [56,58]. Concerns about delaying surgery are not strongly supported by the available data [58]. The evidence base remains limited, however, consisting largely of retrospective studies and small prospective trials without large randomised controlled data in UTUC [56,58]. In current practice, perioperative systemic treatment should be individualised, with particular attention to postoperative renal function, cisplatin eligibility, and the limitations of the existing evidence base.

### 7.5. Immunotherapy

It should be emphasised that most available evidence for immunotherapy in UTUC is derived from broader urothelial carcinoma populations, with only limited UTUC-specific subgroup data. Conclusions for UTUC should therefore be interpreted with appropriate caution.

Immune checkpoint inhibitors (ICIs)—including pembrolizumab, nivolumab, atezolizumab, durvalumab, and avelumab—are now widely used in advanced urothelial carcinoma, with pembrolizumab and atezolizumab supported by phase III randomised trials [13,58]. The CheckMate 274 trial demonstrated that adjuvant nivolumab after radical surgery prolonged DFS in patients with urothelial carcinoma, including a UTUC subgroup comprising approximately 21% of participants. Benefit was most pronounced in patients with PD-L1 expression > 1%, though analyses of renal pelvis and ureteral tumours were limited by small sample sizes [13]. Treatment-related adverse events are common, with fatigue, pruritus, and diarrhoea being most frequent; grade ≥ 3 toxicities—including severe fatigue, anaemia, and liver enzyme elevations—occur less frequently but remain clinically significant [58]. Approximately 12–25% of patients discontinue treatment owing to adverse events [13].

### 7.6. Targeted Therapy

Similarly to immunotherapy, much of the evidence for targeted therapy in UTUC comes from mixed urothelial carcinoma cohorts rather than UTUC-specific studies, and treatment conclusions are therefore partly extrapolated.

Enfortumab vedotin (EV), an antibody–drug conjugate targeting Nectin-4, has demonstrated substantial efficacy in advanced urothelial carcinoma including UTUC. The phase III EV-302 trial demonstrated that the combination of EV with pembrolizumab significantly improved progression-free and overall survival compared with platinum-based chemotherapy, establishing this regimen as a new first-line standard of care in advanced UC [14,56]. UTUC-specific subgroup data from this trial are limited.

**Figure 2:**
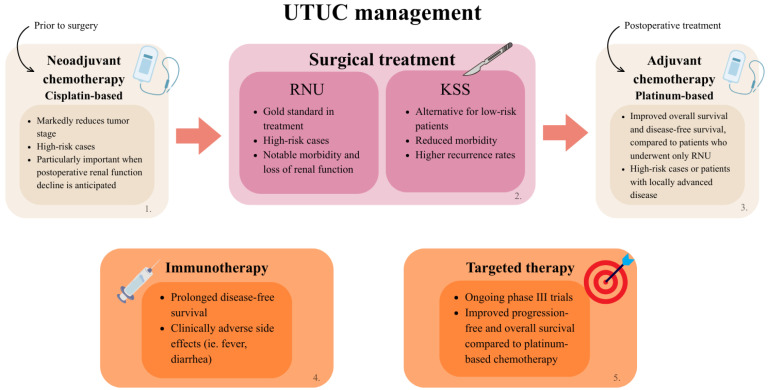
(**1**) Current guidelines support cisplatin-based neoadjuvant chemotherapy prior to nephroureterectomy or ureterectomy, especially when renal function decline is anticipated as it may prevent the use of platinum-based adjuvant chemotherapy. (**2**) Surgical treatment, such as radical nephroureterectomy (RNU) and kidney-sparing surgery (KSS), remains the standard of care in most cases. (**3**) Adjuvant chemotherapy plays an important role in high-risk cases. (**4**) Immunotherapy may prolong disease-free survival, however adverse side effects should be taken into consideration. (**5**) As personalised medicine gains importance, targeted therapy remains an object of research.

FGFR3 mutations are present in a meaningful proportion of UTUC cases, providing a rationale for FGFR-directed therapy. The pan-FGFR inhibitor erdafitinib has demonstrated clinically meaningful benefit in patients with FGFR-altered urothelial carcinoma who have progressed after prior systemic therapy [56]. Emerging biomarkers, including TROP-2, show potential relevance in UTUC: increased TROP-2 expression in low-grade UTUC may carry prognostic and therapeutic implications, whereas Nectin-4, despite broad expression across UC subtypes, is not consistently linked to specific tumour features [14] (Figure 2) (Table 4).

## 8. Discussion

This review summarises current knowledge on the pathophysiology, molecular landscape, and evolving therapeutic strategies for UTUC. Increasing evidence suggests that UTUC is a biologically distinct disease, characterised by unique genomic alterations and tumour microenvironment features that may influence treatment response and clinical outcomes [18,60]. Nevertheless, the majority of clinical evidence guiding current management has been derived from UBC studies, highlighting the need for UTUC-specific research [60,61].

Despite recent advances in systemic therapy, the management of UTUC is still largely based on evidence derived from UBC. Emerging evidence suggests that the distinct molecular landscape of UTUC may translate into differences in therapeutic efficacy [60,61]. Subgroup analyses from clinical trials, including CheckMate 274 and EV-201/EV-301, indicated that the benefit of immune checkpoint inhibitors and antibody–drug conjugates may be less pronounced in patients with UTUC than in those with bladder cancer [62,63]. These findings, together with the higher prevalence of FGFR3 alterations and the predominantly immune-cold phenotype of UTUC, highlight the need for molecularly guided treatment selection and prospective UTUC-specific clinical trials [18,60,61].

Future research in UTUC should focus on three principal areas: biomarker-driven therapeutic strategies, improvement of staging and risk stratification tools, and expansion of multicenter registry-based clinical trials.

**Biomarker-driven therapy.** Predictive biomarkers—including microsatellite instability (MSI), DNA damage repair (DDR) gene deficiencies, tumour mutational burden (TMB), and circulating tumour DNA (ctDNA)—are being evaluated to guide treatment selection and improve patient stratification for immunotherapy and combination regimens [56,64,65]. The clinical value of ctDNA lies in its potential to complement conventional staging by providing molecular information that extends beyond the anatomical assessment of imaging and the limited molecular data obtained from tissue biopsy. If validated in prospective studies, ctDNA could substantially improve risk stratification and guide personalised perioperative treatment strategies in UTUC [66]. Additionally, Padullés et al. [67] stated that postoperative cfDNA levels independently predicted tumour progression and cancer-specific survival after RNU, highlighting the potential of liquid biopsy to identify patients who may benefit from earlier intervention. Available phase II data suggest that patients with MMR alterations achieve higher rates of complete pathological response to ICI combinations, supporting a role for MMR status as a predictive marker in UTUC [56,64]. Moreover, the ongoing phase III PROOF 302 trial demonstrates the growing clinical importance of molecular profiling by evaluating FGFR-targeted therapy in patients with FGFR3-altered urothelial carcinoma, supporting the integration of multi-omics approaches into personalised treatment strategies [68]. Future studies should determine whether integrating minimal residual disease (MRD) assessment and serial molecular profiling into clinical practice can enable adaptive treatment strategies in UTUC [67].

**Staging and risk stratification.** Improved preoperative staging, molecular profiling, and predictive modelling—including AI-assisted risk assessment tools and emerging biomarkers such as ctDNA—are needed to personalise treatment, identify high-risk patients, and optimise perioperative decision-making [66]. AI-assisted risk models may improve risk stratification by integrating clinical, imaging, and molecular data and identifying patterns beyond human interpretation, ultimately supporting more personalised management of UTUC [69]. Key unresolved questions include the optimal use and sequencing of perioperative chemotherapy, the role of lymphadenectomy, and the management of patients with lymphovascular invasion or impaired renal function.

**Registry-based trials.** Given the rarity of UTUC, multicentre collaborations, clinical quality registries, and registry-based randomised controlled trials (RCTs) are essential to facilitate patient recruitment and generate high-quality evidence [66,70]. Such approaches have succeeded in other rare urological malignancies and are well suited to address the evidence gaps that currently limit UTUC management.

**Figure 3:**
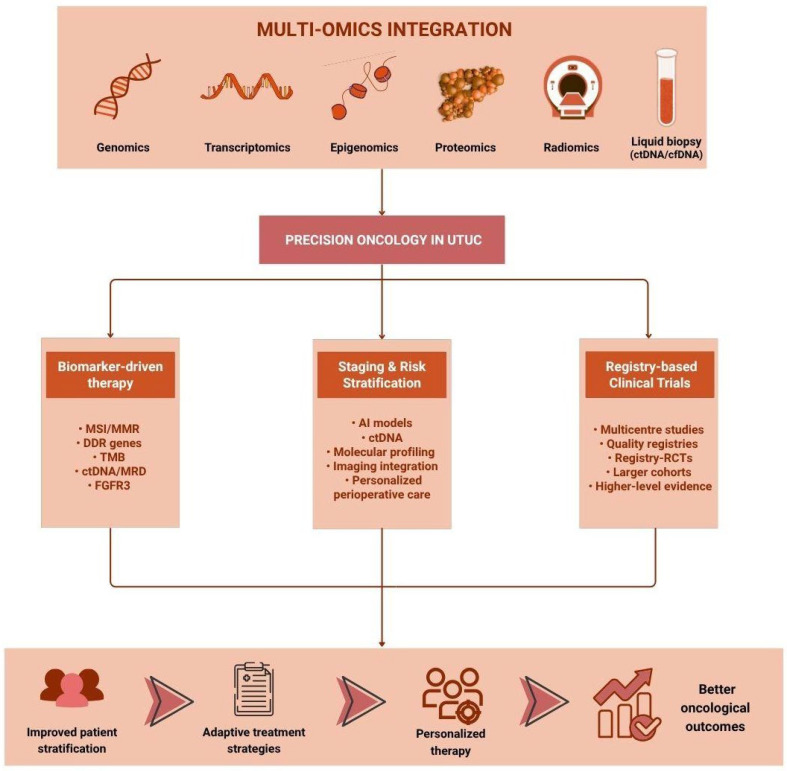
Proposed framework for future research in UTUC. Multi-omics integration enables biomarker-driven therapy, enhanced staging and risk stratification, and multicentre registry-based clinical trials, ultimately supporting precision oncology and improving patient outcomes. *Abbreviations: ctDNA, circulating tumour DNA; cfDNA, circulating free DNA, ctDNA; UTUC, upper tract urothelial carcinoma; MSI, microsatellite instability; MMR, mismatch repair; DDR, DNA damage repair; TMB, tumour mutational burden; AI, artificial intelligence; RCTs, Randomised Controlled Trials*.

Integrating multi-omics approaches into UTUC research may bridge the gap between molecular discoveries and clinical application. These strategies may improve patient stratification, identify novel prognostic and predictive biomarkers, elucidate mechanisms underlying tumour heterogeneity and therapeutic resistance, and accelerate the implementation of precision oncology in clinical practice [71,72] (Figure 3).

## 9. Conclusions

Recent studies have advanced understanding of the molecular background and clinical management of UTUC, confirming that this disease is biologically heterogeneous and not simply a counterpart of bladder urothelial carcinoma. Available evidence supports a role for recurrent molecular alterations, environmental carcinogens such as tobacco smoke and aristolochic acid, and hereditary predisposition—particularly Lynch syndrome—in shaping tumour development and progression.

Important limitations remain. Many mechanistic and therapeutic conclusions still rely on extrapolation from the broader urothelial carcinoma literature, and high-quality UTUC-specific evidence is limited. Surgical treatment remains the cornerstone of management, whereas perioperative chemotherapy, immunotherapy, and targeted therapy are expanding options in selected patients.

The most pressing priority is the generation of UTUC-specific evidence that integrates molecular profiling, environmental exposure data, and clinically meaningful treatment stratification. This approach is essential for improving prevention, risk assessment, and personalised management of this rare but aggressive malignancy.

## Figures and Tables

**Table 1:** Databases and search terms used and number of identified records.

Database	Search Terms	Records Identified
PubMed	‘upper tract urothelial carcinoma’ AND ‘pathogenesis’ AND ‘management’	238
Scopus	‘upper tract urothelial carcinoma’ AND ‘pathogenesis’ AND ‘management’	7
Web of Science	‘upper tract urothelial carcinoma’ AND ‘pathogenesis’ AND ‘management’	107
Embase	‘upper tract urothelial carcinoma’ AND ‘pathogenesis’ AND ‘management’	875

**Table 3:** Summary of carcinogenic mechanisms associated with principal UTUC risk factors.

Risk Factor	Type of Mechanism	Mechanism	Implication	Reference
**Smoking**	DNA damage	4-ABP-DNA adducts	Genotoxic effect	[28,37]
		Mutations in TERT promoter, TP53, KDM6A, FGFR3	Mutations related to carcinogenesis	[43]
		Micronucleated cell	Indicator of cytogenetic damage	[45]
	Enzymatic changes	VEGF-D, COX-2, MMP-9 overexpression	Enhanced lymphangiogenesis and tumour propagation	[40]
**Aristolochic acid**	Enzymatic changes	MMP-2 and MMP-9 overexpression and increased activity; uPA and p-ERK/p-p38 overexpression; TIMP-1 and TIMP-2 decrease	Increased cell migration and invasion	[49]
**Kidney stones**	General cell damage	Epithelial irritation and injury	Oxidative stress and inflammation promoting carcinogenesis	[50,51]
	DNA damage	Concurrent bacterial infections	Possible genotoxic effect via bacterial toxins	[52]

Abbreviations: 4-ABP, 4-aminobiphenyl; VEGF-D, vascular endothelial growth factor D; COX-2, cyclooxygenase-2; MMP-2/-9, matrix metalloproteinase-2/-9; uPA, urokinase-type plasminogen activator; p-ERK, phosphorylated extracellular signal-regulated kinase; TIMP-1/-2, tissue inhibitor of metalloproteinase-1/-2.

**Table 4:** Overview of current and emerging treatment approaches in UTUC.

Drug/Method	Mechanism	Class	Phase	Key Results	Reference
**Radical nephroureterectomy** (RNU)	Removal of kidney, ureter, and bladder cuff	Radical surgery	Approved; gold standard	Best oncologic control in high-risk UTUC; significant morbidity; risk of renal function decline	[10,11,56,57,58,59]
**Kidney-sparing surgery** (KSS)	Localised tumour resection with preservation of renal parenchyma	Organ-preserving surgery	Approved	Comparable CSS in selected patients; higher recurrence rates; lower morbidity	[10,11,57,58,59]
**Gemcitabine + cisplatin/carboplatin**	DNA crosslinking (platinum) + inhibition of DNA synthesis (gemcitabine)	Adjuvant chemotherapy	Approved	POUT trial: DFS HR 0.45 (3-year: 71% vs. 46%); cisplatin superior to carboplatin	[11,12,13,56,58]
**Cisplatin-based neoadjuvant therapy**	Pre-surgical cisplatin-based regimen; both kidneys retained, increasing cisplatin eligibility	Neoadjuvant chemotherapy	Approved	~60% downstaging; 14–19% complete response; improved OS and node-negative disease	[13,58]
**Nivolumab**	PD-1 receptor inhibition	Immunotherapy (ICI)	Approved	CheckMate 274: prolonged DFS after radical surgery; UTUC subgroup ~21%; benefit strongest in PD-L1 > 1%	[13]
**Enfortumab vedotin** (EV)	Antibody–drug conjugate targeting Nectin-4	Targeted therapy (ADC)	Approved	Effective in advanced UC including UTUC; evidence largely from mixed UC cohorts	[14,55]
**EV + pembrolizumab**	Nectin-4 targeting (EV) + PD-1 inhibition (pembrolizumab)	Combination targeted/immunotherapy	Phase III	EV-302: improved PFS and OS vs. platinum-based chemotherapy; new first-line standard of care in UC	[14,55]
**Erdafitinib**	Pan-FGFR inhibitor	Targeted therapy	Phase III	Clinically meaningful benefit in FGFR3-altered UC progressing after prior systemic therapy	[56]

Abbreviations: RNU, radical nephroureterectomy; KSS, kidney-sparing surgery; DFS, disease-free survival; HR, hazard ratio; OS, overall survival; ICI, immune checkpoint inhibitor; PD-1, programmed cell death protein 1; EV, enfortumab vedotin; UC, urothelial carcinoma; UTUC, upper tract urothelial carcinoma; CSS, cancer-specific survival; FGFR, fibroblast growth factor receptor; ADC, antibody–drug conjugate; PFS, progression-free survival.

## Data Availability

No new data were created or analysed in this study. Data sharing is not applicable.
